# Three Cases of Occupational Allergic Contact Dermatitis Where Causative Agents Were Identified Using Patch Test Panel S

**DOI:** 10.7759/cureus.45891

**Published:** 2023-09-25

**Authors:** Naoki Sasaki, Natsuko Saito-Sasaki, Ken Washio, Kaoru Takayama, Yu Sawada

**Affiliations:** 1 Dermatology, University of Occupational and Environmental Health, Kitakyushu, JPN; 2 Dermatology, Kobe City Nishi-Kobe Medical Center, Kobe, JPN; 3 Dermatology, Saiseikai Kawaguchi General Hospital, Kawaguchi, JPN

**Keywords:** patch test panel s, skin, patch test, allergy, occupational contact dermatitis

## Abstract

Identifying the causative substances of occupational contact dermatitis is challenging because of several chemicals and materials in the workplace that can cause contact dermatitis. We experienced three cases of intractable eczema identified as work-related contact dermatitis by Patch Test Panel S, which helped identify the possible substances. We experienced three cases of occupational allergic contact dermatitis, and their causative agents were identified by Patch Test Panel S. Although there are some limitations, Patch Test Panel S might be useful to determine the substrates to cause allergic contact dermatitis in occupational scenarios.

## Introduction

In the clinical setting, identifying the causative substances of occupational allergic contact dermatitis is challenging because of several chemicals and materials in the workplace that can cause contact dermatitis [1,2]. On the contrary, various dermatologists face difficulties in determining the causative substrate of allergic contact dermatitis in occupational scenarios. In those cases, patch testing is one of the tools to figure out the cutaneous allergic factor in workers.

Various causal allergic agents are placed in the occupational scenario. In addition, workers face many chances to be exposed to an abundance of these materials. This is one of the difficulties in figuring out the causative agents of allergic contact dermatitis. Therefore, a comprehensive patch-testing panel tool is necessary to determine the agents. The Japan Baseline Series 2015 (JBS 2015) is a modified series, omitting allergens with low sensitization rates in Japan from the T.R.U.E. test (Smartpractice Denmark, Hillerød, Denmark), and consists primarily of Patch Test Panel S, a simplified T.R.U.E. test that contains 22 types of allergens [3]. However, no case series report has been reported to describe the importance of Patch Test Panel S, and how it is crucial to identify the allergic agents. Herein, we experienced three cases of intractable eczema identified as work-related contact dermatitis by Patch Test Panel S, which helped identify the possible substances.

## Case presentation

Case 1

**Figure 1:**
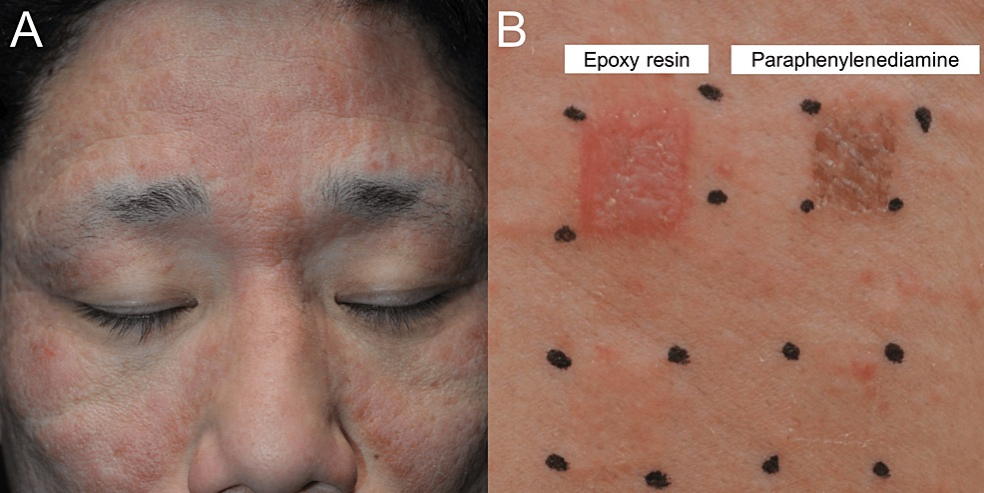
Clinical manifestations and patch testing in Case 1 Clinical manifestation (A) and patch testing (B) in Case 1. (A) Itchy and scaly erythematous plaques were located on his face. (B) Patch Test Panel S showed positive reactions to epoxy resin and paraphenylenediamine.

A 26-year-old man observed intractable itchy erythema on his face and axilla, and topical steroids were ineffective. Because his skin eruptions were limited to his face, axilla, and hands (Figure 1A), we speculated that his skin eruptions were due to allergic contact dermatitis (ACD), photosensitivity dermatitis, or drug eruption. However, he did not use any medications or dietary supplements. In addition, his skin eruption was not located on the V-neck zone. Since the patient did not know what substances could have caused his dermatitis, Patch Test Panel S was conducted to determine the possible causative substances. This patch testing directly showed positive reactions to epoxy resin and paraphenylenediamine (Figure 1B). After a re-examination through an interview, the patient was engaged in affixing sheets to prevent concrete deterioration on expressways and wore rubber gloves at work without face personal protection equipment. Furthermore, this task involved using adhesives and rubber gloves. His skin eruptions improved after not using these adhesives and rubber gloves and his skin eruption did not entirely recur.

Case 2

**Figure 2:**
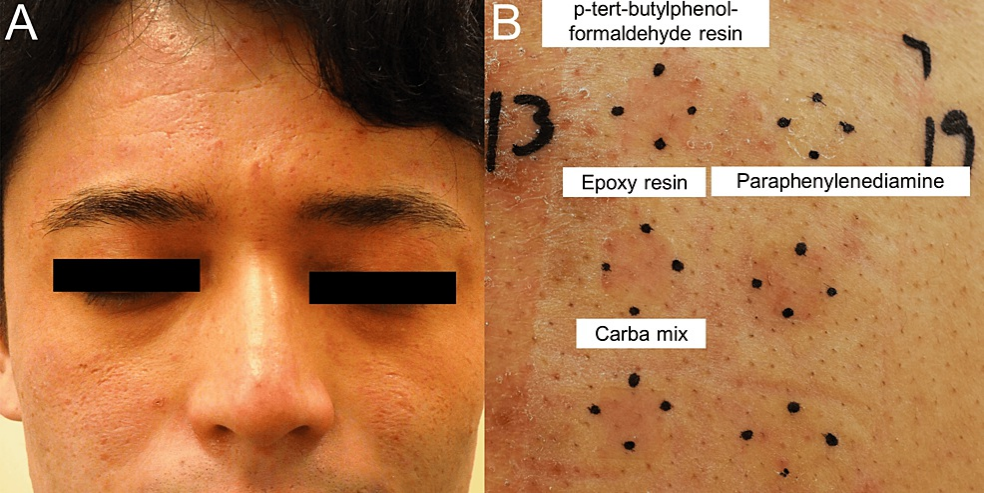
Clinical manifestation and patch testing in case 2. Clinical manifestation (A) and patch testing (B) in case 2. (A) Scaly erythematous plaques were located on his face. (B) Patch Test Panel S identified positive reactions to p-tert-butylphenol-formaldehyde resin, epoxy resin, paraphenylenediamine, and carba mix.

A 25-year-old man experienced intractable scaly erythema on his face and hands, which were not improved by the topical application of steroids (Figure 2A). His skin eruptions worsened for six months. He had also recently started his current work of painting nine months ago. Therefore, he thought that his skin eruption might be associated with his work. However, he could not recognize what substrate was a causative agent for his skin eruption. He was referred to our department for an evaluation of his skin eruptions. Patch Test Panel S identified positive reactions to p-tert-butylphenol-formaldehyde resin, epoxy resin, paraphenylenediamine, and carba mix. The patient’s work involved painting using toluene, xylene, benzene, and formaldehyde and wearing rubber gloves containing these positive allergens (Figure 2). The painting materials and rubber gloves were thought to have triggered his ACD. He wasn't wearing a face mask. After the patient changed work, his skin eruptions improved without recurrence.

Case 3

**Figure 3:**
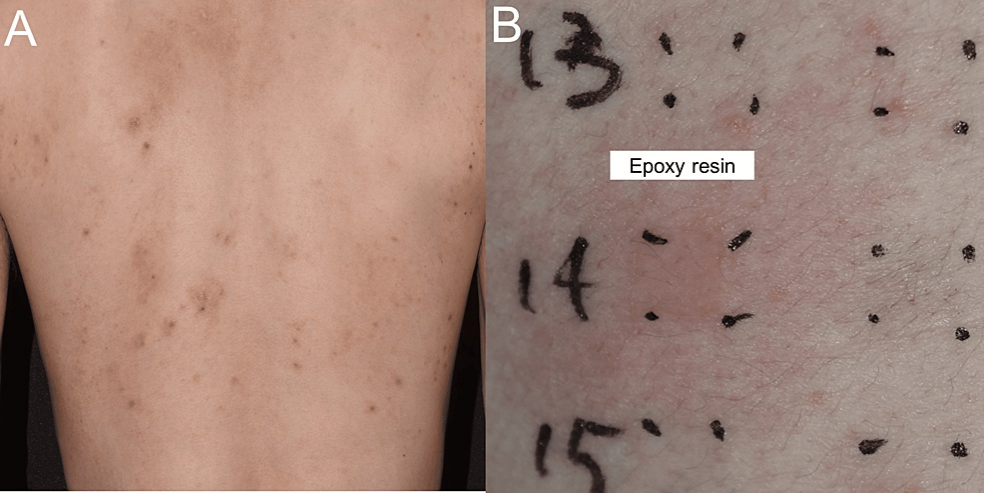
Clinical manifestation and patch testing in Case 3 Clinical manifestation (A) and patch testing (B) in Case 3 (A) Erythematous eruption was observed on his trunk. (B) Patch Test Panel S identified positive reactions to epoxy resin.

A 32-year-old man noticed an itch and erythematous eruption on his trunk and extremities for three months, which was intractable to topical steroids and anti-histamine drugs (Figure 3A). He was referred to our department. Patch Test Panel S identified positive reactions to epoxy resin. The patient was working with spray paints in an industrial motor manufacturing company. The coating substances used in spray painting contained epoxy resin, indicating that the coating spray painting materials containing epoxy resin were allergens for his skin eruptions (Figure 3B). After the patient switched to work not using spray paint, his skin eruption completely improved.

## Discussion

The traditional conventional patch testing requires that aqueous or Vaseline-based allergen reagents be placed in a Finn Chamber one at a time, which makes it difficult to maintain a uniform dose or concentration, especially in Vaseline-based allergens used in patch testing. The T.R.U.E. TEST® (Thin-layer Rapid Use Epicutaneous TEST: TT) system was developed in 1984 by Fischer and collaborators at Uppsala University in Sweden to solve these problems [4]. A polyester supplementation is coated with an allergen that has been dissolved in a hydrophilic base to create a surface of patch testing. Among several patch test panels that have been used in the world [5], Patch Test Panel S covers 22 materials, referred to as the JBS 2015.

Although this is a comprehensive representation of allergens used in patch testing, this examination might cause an unnecessary chance to obtain sensitization causing allergic contact dermatitis. Previous studies showed a possible increased risk of sensitization in several substrates [5,6]. Therefore, these findings might suggest recommending a case of allergic contact dermatitis to use Patch Test Panel S.

As previously indicated, patch testing is useful for the diagnosis and identification of occupational contact dermatitis [7]. A study showed that 8.9% of occupational workers were highly sensitized to an epoxy component; however, 25% of occupational workers sensitized to epoxy components showed a negative reaction to the epoxy resin by the TRUE test® panel [8], suggesting a possibility of a false negative result by this patch test panel.

Furthermore, Patch Test Panel S does not completely cover the 24 allergens of JBS 2015 such as mercuric chloride and urushiol. Therefore, clinicians should keep in mind the limitations of this examination although it is convenient and easy to apply for occupational allergic contact dermatitis patients.

For occupational skin diseases, identifying the causative agents and subsequently considering relocation of workplaces or avoiding contact with allergens should be decided by occupational physicians and dermatologists to improve the skin condition of employees. To help identify the causative agent of occupational ACD, Patch Test Panel S contains representative allergic candidates and may be useful in identifying the specific allergen in intractable occupational contact dermatitis cases, leading to the further investigation of actual allergic causes in the occupation places. Fortunately, the causative agents were identified by Patch Test Panel S as previously described in our cases [3].

## Conclusions

We present a case series of occupational contact dermatitis, which was identified by Patch Test Panel S. Suspected cases of occupational contact dermatitis might be a candidate for Patch Test Panel S to identify allergic agents in the occupational scenario. Although conventional patch testing is useful for arranging specific allergen tests, Patch Test Panel S might be convenient for performing type IV cutaneous allergic reaction tests on the skin.

## References

[1] Sawada Y (2023). Occupational skin dermatitis among healthcare workers associated with the COVID-19 pandemic: a review of the literature. Int J Mol Sci.

[2] Karagounis TK, Cohen DE (2023). Occupational hand dermatitis. Curr Allergy Asthma Rep.

[3] Ito A, Suzuki K, Matsunaga K (2022). Patch testing with the Japanese baseline series 2015: a 4-year experience. Contact Dermatitis.

[4] Fischer TI, Maibach HI (1985). The thin layer rapid use epicutaneous test (TRUE-test), a new patch test method with high accuracy. Br J Dermatol.

[5] Suzuki K, Yagami A, Ito A, Kato A, Miyazawa H, Kanto H, Matsunaga K (2019). Positive reactions to gold sodium thiosulfate in patch test panels (TRUE Test) in Japan: a multicentre study. Contact Dermatitis.

[6] Hillen U, Jappe U, Frosch PJ (2006). Late reactions to the patch-test preparations para-phenylenediamine and epoxy resin: a prospective multicentre investigation of the German Contact Dermatitis Research Group. Br J Dermatol.

[7] Li LF, Sujan SA, Wang J (2003). Detection of occupational allergic contact dermatitis by patch testing. Contact Dermatitis.

[8] Christiansen AG, Carstensen O, Sommerlund M (2022). Prevalence of skin sensitization and dermatitis among epoxy-exposed workers in the wind turbine industry. Br J Dermatol.

